# The Role of Major Salivary Gland Ultrasound in the Diagnostic Workup of Sicca Syndrome: A Large Single-Centre Study

**DOI:** 10.3390/tomography10010006

**Published:** 2024-01-08

**Authors:** Giulia Vallifuoco, Paolo Falsetti, Marco Bardelli, Edoardo Conticini, Stefano Gentileschi, Caterina Baldi, Suhel Gabriele Al Khayyat, Luca Cantarini, Bruno Frediani

**Affiliations:** Rheumatology Unit, Department of Medical Sciences, Surgery and Neurosciences, University of Siena, 53100 Siena, Italy; paolo.falsetti@virgilio.it (P.F.); marco.bardelli@ao-siena.toscana.it (M.B.); conticini@student.unisi.it (E.C.); stefano.gentileschi@unisi.it (S.G.); catebaldi3@gmail.com (C.B.); alkhayyat@student.unisi.it (S.G.A.K.); cantarini@unisi.it (L.C.); bruno.frediani@unisi.it (B.F.)

**Keywords:** salivary glands ultrasound, labial salivary glands biopsy, sicca syndrome, Sjogren syndrome

## Abstract

(1) Objective: To determine the diagnostic accuracy of major salivary gland ultrasonography (SGUS) in primary Sjogren’s syndrome (SS), we used the Outcome Measures in Rheumatology Clinical Trials (OMERACT) scoring system on a large single-centre cohort of patients with sicca syndrome. (2) Method: We retrospectively collected the clinical, imaging and serological data of all the patients referred with a suspicion of SS who underwent SGUS and minor salivary glands biopsy. (3) Results: A total of 132 patients were included. The SGUS scores were correlated between the two sides (*p* < 0.001). The diagnostic cut-off for SS (AUROC: 0.7408) was 6 for the SGUS-global sum (sensitivity: 32.43%; specificity: 96.84%). The cut-off with the highest specificity for SS diagnosis was 7. In the patients with a final diagnosis of SS, the mean SGUS score was significantly higher (*p* < 0.001) than that of the non-SS patients (3.73 vs. 1.32 for the SGUS-global sum). A significant correlation was demonstrated between the SGUS scores and final SS diagnosis (*p* < 0.001), biopsy positivity (*p* < 0.001), ANA positivity (*p* = 0.016), Ro-SSA positivity (*p* = 0.01), and gland fibrosis (*p* = 0.02). (4) Conclusions: SGUS, using the OMERACT scoring system, has moderate sensitivity and high specificity for the diagnosis of SS. The scoring showed a strong and direct correlation with all the clinical hallmarks of SS diagnosis, such as the positivity of a labial salivary gland biopsy, ANA and Ro-SSA statuses, and salivary gland fibrosis. Because of its high specificity, a SGUS-global score > 6 could be therefore employed for the diagnosis of SS in the case of ANA negativity or the unavailability of a biopsy.

## 1. Introduction

Sicca syndrome is a condition characterised by the symptomatic dryness of the mucous membranes, in particular the ocular (xerophthalmia) and oral ones (xerostomia). Sicca syndrome affects up to 30% of the population that is over 65 years of age [1], but there are different causes. 

The most common autoimmune condition causing sicca syndrome is primary Sjogren syndrome (SS), characterised by inflammation and the lymphocytic infiltration of the exocrine glands, eventually leading to progressive parenchymal destruction and atrophy [2]. One third of the patients exhibit a systemic extra glandular manifestation, such as B-cell lymphomas, skin vasculitis, arthritis, interstitial lung disease, and renal failure [3]. 

Several other systemic diseases, as well as drugs, may also be associated with SS through hyposecretion or exocrine gland destruction [4]. Finally, oral and/or ocular dryness can be referred to by the patients, without any objective evidence of glandular damage or hypofunction.

The American College of Rheumatology (ACR) and the European League against Rheumatism (EULAR) classification criteria, which still have a paramount role in the diagnosis of SS, include both a serological (presence of anti-SSA antibodies) and a histological domain (evidence of lymphocytic sialadenitis on a labial gland biopsy) [5]. Nevertheless, although it is not mentioned in ACR/EULAR criteria, salivary gland ultrasonography (SGUS) has been proven to display a strong correlation with clinical or serologic data, as well as between different US scores, therefore arising as a promising tool in the diagnostic workup of SS [6,7,8,9,10]. In order to standardise SGUS, the Outcome Measures in Rheumatology Clinical Trials (OMERACT) group has proposed an expert consensus definition of SGUS elementary lesions and a new four-grade scoring system (Grades 0–3) for diagnosing salivary gland involvement in SS [11].

The aim of our work was to retrospectively evaluate the diagnostic performance of SGUS (using the new OMERACT scoring system) for SS in a large single-centre cohort of patients with sicca syndrome who completed a full diagnostic pathway ending with a minor salivary glands biopsy in the scenario of a real clinical practice. The novelty of this study is the availability of all the patients who have undergone a labial salivary biopsy to be used as a diagnostic gold standard for SS final diagnoses. 

Our secondary endpoint was to study the potential role of SGUS to detect the other causes of sicca syndrome (dysmetabolic conditions, ageing, and drug-induced and subjective dryness), suggesting the best performing diagnostic pathway for the patients referred with sicca syndrome. 

## 2. Materials and Methods 

### 2.1. Patients and Methods

The study retrospectively included all patients referred in our Rheumatology Unit from January 2021 to September 2022 with suspected SS. 

All patients were aged ≥18 years, and they underwent a complete diagnostic workup for sicca syndrome in order to diagnose SS according to the ACR/EULAR 2016 criteria [5] and to exclude secondary causes. All subjects underwent at least a Schirmer test for functional testing, autoimmunity testing, SGUS, and a minor salivary gland biopsy. 

### 2.2. Clinical, Serological, and Immunological Assessments 

The clinical evaluation included assessments of the following parameters: ocular symptoms, oral symptoms, or both ocular signs (the Schirmer test result was defined positive if ≤5 mm after 5 min and borderline if it was between 5 and 10 mm after 5 min). Moreover, all patients completed a questionnaire, where past pathological and pharmacological anamnesis were investigated. In particular, patients were asked about their smoking habits, possible head or neck radiotherapy, dental prostheses, and disease duration (defined as duration of symptoms of dryness; short disease duration is <3 years and long disease duration is >3 years) [12].

All patients underwent serological tests searching for anti-nuclear antibodies (ANAs), anti-extractable nuclear antigens (ENAs), anti-double-stranded DNA (dsDNA) antibodies, and rheumatoid factor (RF) IgG, as well as routine ones. ANAs were detected by indirect immunofluorescence on Hep2 cells (Euroimmune, Lubeck, Germany), ENAs were detected using both a fluorescent enzyme immunoassay (FEIA-Thermofisher) and Western blot (WB, ANRIKA). Even if the dsDNA antibodies and RF were assessed for clinical aims, the results are not available for this study.

### 2.3. Salivary Gland Ultrasonography 

All patients underwent SGUS examination of major salivary glands (MSG) performed by an expert rheumatologist sonographer (20 years of experience in musculo-skeletal and rheumatologic US) who was blind to the clinical data. SGUS was conducted one hour before biopsy.

Each patient underwent SGUS bilateral examination of parotid and submandibular glands.

**Figure 1:**
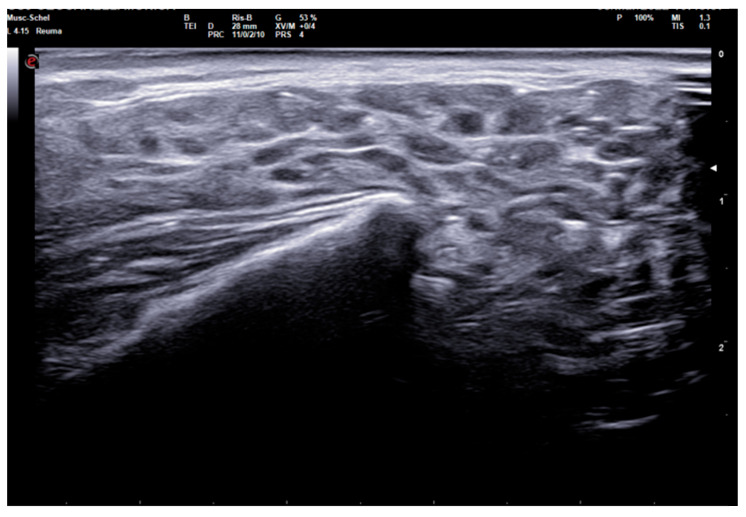
Parotid SGUS score 3 in primary Sjogren syndrome. Female 51-year-old patient with sicca syndrome, positive Schirmer test, positive autoimmunity (ANA+, Ro-SSA+), positive labial biopsy for SS. A coronal trasverse scan over left parotid (4–15 MHz linear probe) shows diffuse inhomogeneity with hypo-anechoic areas occupying the entire gland volume, surrounded with septal echoic thickening and scanty normal parenchymal tissue (SGUS-OMERACT score = 3).

The 0–3-graded OMERACT score was used to assess the ultrasonographic structure of each gland [13,14,15]. The score was applied on each gland, and defined as follows: Grade 0, normal parenchyma (sono-structure comparable to the thyroid gland); Grade 1, mild inhomogeneity without anechoic or hypoechoic areas and hyperechogenic bands; Grade 2, moderate inhomogeneity with focal anechoic or hypoechoic areas; and Grade 3, severe inhomogeneity with diffuse anechoic or hypoechoic areas occupying the entire gland surface [13] (Figure 1). 

Moreover, for each gland, adipose (homogeneously hyperechoic compared with adjacent tissue, and incomplete definition of deep margin of the gland) and fibrous involution (hyperechoic bands that develop into fibrotic tissue indistinguishable from the adjacent soft tissues) and the number of intra-parenchymal lymph nodes were recorded [11]. SGUS was performed using a 6–18 MHz linear probe, MyLab Twice (Esaote, Genoa, Italy), from January 2021 to January 2022, and a 4–15 MHz or 8–24 MHz linear probe, MyLab X8 eXP Esaote (Esaote, Genoa, Italy) from January 2022, with standardised B-mode settings optimised for all examinations. The examination started with a medium setting of time gain compensation and a gain between 50% and 80%. The parameters varied to achieve a better visualisation of deeper margin of the parotid glands.

### 2.4. Minor Salivary Gland Biopsy

All patients underwent a biopsy of the minor salivary glands using the punch technique proposed by Guevara-Gutierrez [16] under local anaesthesia with lidocaine. A 4 mm punch and 4-0 absorbable sutures were used. Salivary gland samples were subsequently fixed in formalin and evaluated using the Focus Score (FS) method by the Pathology Department of Siena. A histological report was considered diagnostic for SS when FS ≥ 1 [17]. We excluded from the evaluation all invalid histological reports, defined as those with total surface area obtained of < 4 mm^2^.

### 2.5. Statistical Analysis

Data were expressed as mean ± standard deviation or percentages. For categorical variables, Chi-squared or Fisher’s exact tests were used to compare proportions between groups. Student’s *t*-test was used to compare the means of continuous variables between two groups when the distribution of data were normal, and with Welch’s correction otherwise.

A non-parametric Kruskal–Wallis test was used to compare the means of continuous variables among categorical groups, while a Dwass–Steel–Critchlow–Fligner (DSCF) test was used for pairwise comparisons. Non-parametric Spearman rank and Kendall Tau-B tests were applied in order to correlate continuous or categorical variables.

Multivariable linear regression was performed with all significant variables entered in a stepwise way to identify which factors independently correlated with the dependent variable, and this was checked for multicollinearity.

Binomial logistic regression and receiver operating characteristic (ROC) curve analy-sis were used to determine the diagnostic performances of each SGUS score (both sum of parotid and submandibular gland scores on a single side and sum of all four gland scores) in detecting SS; final clinical diagnosis was used as the gold standard reference. The validity of each SGUS score for diagnosis of SS was determined by an estimation of the sensitivity and specificity of various cut-off points of SGUS, and the area under the ROC curve (AUROC) was calculated for each SGUS score. Youden’s J-statistic method was applied to obtain the optimal cut-off values.

Further frequencies analysis of both biopsy and SGUS results was performed dividing the sicca syndrome population into three clinical phenotypes as suggested in previous work [4]: Group 1 = subjective sicca syndrome (positive subjective symptoms, negative functional testing and autoimmunity), Group 2 = objective non-autoimmune sicca syndrome (positive subjective symptoms and functional testing, negative autoimmunity), Group 3 = objective autoimmune sicca syndrome (positive subjective symptoms, functional testing, and autoimmunity). 

The feasibility of SGUS was assessed by measuring the time spent by the operator and evaluating the comfort experienced by the patient during examination.

The level of statistical significance was set at a *p*-level of 0.05. Statistical analyses were performed using Jamovi 1.6.16 and GraphPad 9.5.0 statistical packages. 

### 2.6. Ethics

The study was conducted in accordance with the tenets of the Declaration of Helsinki, and the use of clinical data for research purposes was approved by the local Ethics Committee of the University of Siena (Reference No. 22271, “RHELABUS”). Written informed consent was obtained for all the procedures according to the local Institutional review board guidelines. 

## 3. Results

### 3.1. Participants Characteristics and Descriptive Statistics 

A total of 132 subjects with sicca syndrome completed the diagnostic pathway for SS (female/male ratio 124/8, age in years 57.2 ±SD 13.2). A final diagnosis of SS was made in 37 patients (28%), fulfilling the ACR/EULAR criteria (female/male ratio 33/4, age in years 57.4 ±SD 14.7) [5]. No patient was diagnosed with secondary Sjogren syndrome.

Demographic, anthropometric, and clinical characteristics of participants are summarised in Table 1.

When patients were subdivided based on the definite diagnosis (SS vs. non-SS), no statistically significant differences were observed in terms of age and gender (*p* = 0.917 and 0.156, respectively) nor for Schirmer test (*p* = 0.355). Long disease duration (>3 years of dryness symptoms) was reported in 15/37 (40.5%) of SS patients.

### 3.2. Symmetrical MSG Involvement on SGUS and among Different Glands

SGUS scores correlated well between the two sides. Right parotid gland SGUS correlated with the left one (r = 0.796, *p* < 0.001); right submandibular gland SGUS correlated with the left one (r = 0.768, *p* < 0.001).

Similarly, the sum of SGUS scores of the right side significantly correlated with the left side one (r = 0.788, *p* < 0.001).

Additionally, the mean of SGUS scores between the two sides did not show significant differences.

**Figure 2:**
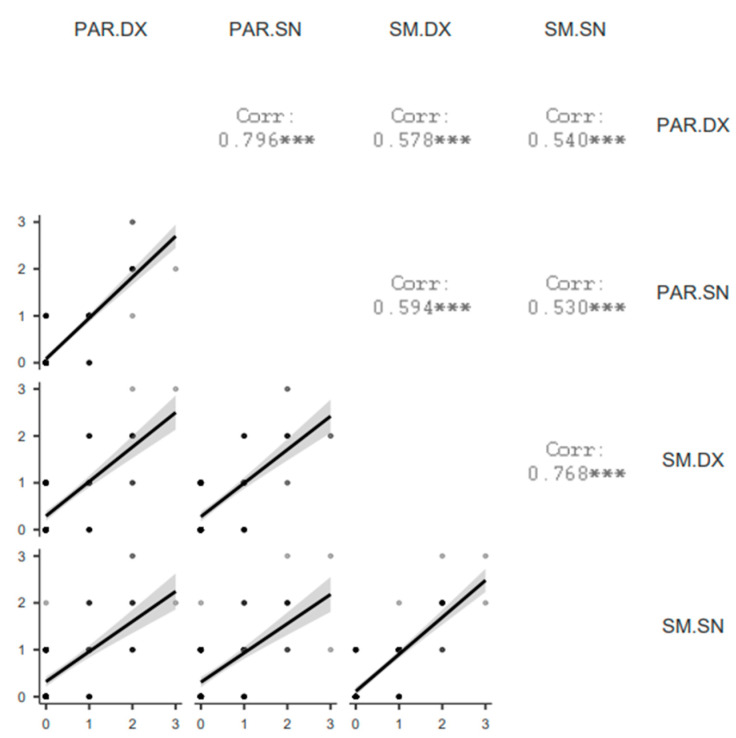
Correlation matrix plot of SGUS scores among glands. PAR = parotid gland, SM = submandibular gland, DX = right, SN = left. *** = *p* < 0.001.

A lower (Spearman rho between 0.530 and 0.594) but significant correlation (*p* < 0.001) was demonstrated among different glands (parotids vs. submandibular) (Figure 2).

### 3.3. Diagnostic Performance of SGUS

**Figure 3:**
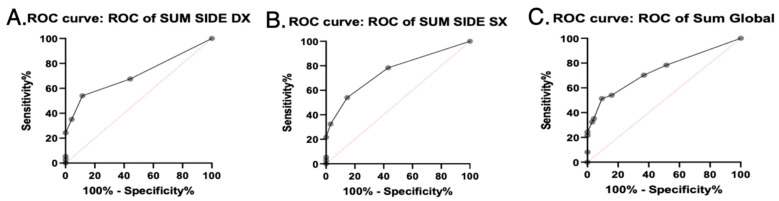
SGUS AUROC curves. Area under ROC curves of SGUS scores with clinical relevance. (**A**) Sum side DX = sum of SGUS scores of right parotid and submandibular glands. (**B**) Sum side SX = sum of SGUS scores of left parotid and submandibular glands. (**C**) Sum global = sum of SGUS scores of all four glands.

Binomial regression analysis was performed to obtain diagnostic cut-offs of SGUS for the diagnosis of SS (Figure 3). 

For the SGUS-right side, the AUROC curve was estimated at 0.7112 (SE 0.05678, 95% CI 0.6–0.8225, *p* = 0.0002). The diagnostic cut-off obtained with Youden J-statistic was 3 (sensitivity 35.14%, specificity 95.79%, likelihood ratio 8.345). The cut-off with the higher specificity for SS diagnosis was 4 (sensitivity 24.32%, specificity 100%).

For the SGUS-left side, the AUROC curve was estimated at 0.7538 (SE 0.05067, 95% CI 0.6545–0.8531, *p* < 0.0001). The diagnostic cut-off obtained with Youden J-statistic was 3 (sensitivity 32.43%, specificity 96.84%, likelihood ratio 10.27). The cut-off with the higher specificity for SS diagnosis was 4 (sensitivity 21.62%, specificity 100%).

For SGUS-global, the AUROC curve was estimated at 0.7408 (SE 0.05299, 95% CI 0.637–0.8447, *p* < 0.0001). The diagnostic cut-off determined with Youden J-statistic was 6 (sensitivity 32.43%, specificity 96.84%, likelihood ratio 10.27). The cut-off with the higher specificity for SS diagnosis was 7 (sensitivity 24.32%, specificity 100%). 

### 3.4. Differences on SGUS between SS and Non-SS Patients

**Figure 4:**
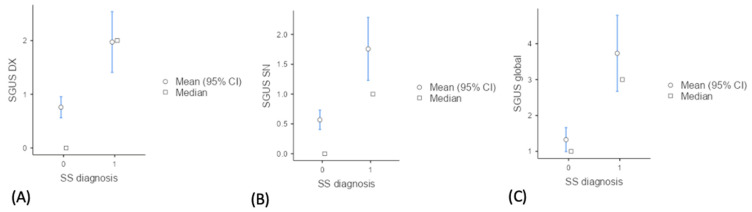
Descriptive plots of mean SGUS scores in SS vs. non-SS. (**A**) Distribution of SGUS scores for the right parotid and submandibular glands (SGUS DX), (**B**) left parotid and submandibular glands (SGUS SN) (**C**) and the sum of all four glands (SGUS global) between SS (primary Sjogren syndrome) and non-SS groups.

In patients with a final diagnosis of SS, the mean SGUS scores were significantly higher (*p* < 0.001) than in those of non-SS patients (1.97 vs. 0.79 for right side SGUS, 1.75 vs. 0.56 for left side SGUS, 3.73 vs. 1.32 for global sum SGUS); see Table 1 and Figure 4.

A significant correlation was demonstrated between SGUS scores and final SS diagnosis (*p* < 0.001). The correlation was maintained (*p* < 0.001) when adjusting SGUS scores for age or gender. Glandular fibrosis significantly correlated to final SS diagnosis (*p* = 0.019), independently from age.

A significant presence of fibrosis on SGUS was evidenced in SS (*p* = 0.035), while no differences emerged for the incidence of adipose gland involution and the number of intra-parenchymal lymph nodes. The adipose gland involution and the number of intra-parenchymal lymph nodes showed significant correlation (r = 0.382, *p* = 0.006), also when controlled for age and gender (r = 0.346, *p* = 0.016).

### 3.5. Correlations among SGUS Scores and Anthropometric, Clinical, Laboratory Parameters

In the overall population, SGUS global (and single-sided) scores significantly correlated with final diagnosis (*p* = 0.001), biopsy positivity (*p* < 0.001), ANA positivity (*p* = 0.015), Ro-SSA positivity (*p* = 0.009), and gland fibrosis (*p* = 0.019). A trend of correlation was evidenced between SGUS scores and the number of intra-parenchymal lymph nodes (*p* = 0.07).

The adipose gland involution showed significant correlation with age (r = 0.221, *p* = 0.011) and the number of intra-parenchymal lymph nodes (*p* = 0.006). Gland fibrosis showed significant correlation with SS diagnosis (*p* = 0.003), biopsy positivity (*p* = 0.002), age (*p* = 0.026), and SGUS scores (*p* = 0.019).

In the overall population, a model of backward stepwise multivariate regression analysis showed that the variability of adipose gland involution is mostly dependent on age, disease duration and number of intra-parenchymal lymph nodes (r^2^ = 0.469, *p* = 0.009). Similarly, the evidence of gland fibrosis largely depends on global SGUS scores, age and number of medications in therapy (r^2^ = 0.277, *p* = 0.017).

A diagnostic SGUS score (SGUS global sum > 6, diagnostic for SS) was more frequent in patients with long disease duration (*p* = 0.011), even if no clear correlation emerged between disease duration and SGUS scores on the overall sicca syndrome population.

### 3.6. Application of SGUS in Real Clinical Practice

Contingency analysis of the principal diagnostic steps (expressed as a dichotomous variable) was performed in order to compare diagnostic capabilities of each diagnostic step for the final diagnosis of SS. 

Among anamnestic information about sicca syndrome disease, only disease duration of longer than 3 years showed an OR of 12.5 (*p* < 0.001, 95% CI 2.41–64.5) for SS diagnosis. 

Schirmer test positivity showed a non-significant OR (1.51). 

ANA positivity showed an OR of 2.58 (*p* < 0.018, 95% CI 1.19–5.62) and Ro-SSA positivity showed an OR of 4.92 (*p* < 0.001, 95% CI 2.08–11.6) for SS diagnosis. 

SGUS positivity, with a best performing cut-off (≥6), showed an OR of 14.7 (*p* < 0.001, 95% CI 3.85–56.2), whereas SGUS positivity, with highest specificity cut-off (≥7) and an OR of 63.7 (*p* < 0.001, 95% CI 3.59–1128) for SS diagnosis. 

Labial biopsy positivity showed the highest OR of 4648 (*p* < 0.001, 95% CI 185–116,701, Haldane–Ascombe correction applied) for SS diagnosis.

SGUS and labial biopsy, considered as second-level diagnostic steps, were then applied and compared when the overall population was divided ino three clinical phenotype groups (see Methods for details): Group 1 = subjective sicca syndrome (34 patients), Group 2 = objective non-autoimmune sicca syndrome (79 patients), Group 3 = objective autoimmune sicca syndrome (19 patients). Table 2 shows the frequencies of positivity of biopsy and SGUS among these clinical phenotype groups. Both SGUS positivity (with best performing cut-off ≥ 6) and labial biopsy showed significant differences among the groups (*p* = 0.009 and *p* = 0.002, respectively). 

### 3.7. Feasibility of SGUS in the Real Clinical Practice

A time of 5 min was sufficient for setting US parameters and completing the grey-scale US examination of MSG. No adverse events occurred during examinations, and all patients considered this examination quick, not painful, and mostly comfortable.

## 4. Discussion

The aim of our work was to retrospectively evaluate the diagnostic performance of the SGUS OMERACT scoring system for SS in a large single-centre cohort of patients with sicca syndrome, in a scenario of real clinical practice. The availability in all the patients of labial salivary biopsy, used as diagnostic gold standard for SS, represents a difference from previous works adopting this scoring system [14,15], in which labial biopsy was not available for all patients.

Our secondary endpoint was to study the potential role of SGUS to detect other causes of sicca syndrome (dysmetabolic condition, ageing, drug-induced and subjective dryness), suggesting the best performing diagnostic pathway of patients referring with sicca syndrome. 

Our large, single-centre, retrospective study proved that US can be considered an excellent diagnostic tool to discriminate between SS and symptomatic sicca syndrome patients with the new OMERACT scoring system, with a significant AUROC of 0.7408. 

Significant differences in SGUS scores were obtained between SS and non-SS patients according to previous recent works adopting the same scoring method [14,15].

ROC analysis showed that the optimal SGUS cut-offs of 6 for the global sum of four glands and 3 for the unilateral side sum are comparable, although they are lower than those reported in previous recent studies [14,15]. This difference could be related to a shorter disease duration and different gold standard of our study compared to previous works [14,15].

Nevertheless, the optimal SGUS cut-off for each single gland was 2, substantially equal to a previous report [14,15]. These data confirm that the OMERACT SGUS score is a reliable and repeatable scoring system, with high concordance among different centres. 

**Figure 5:**
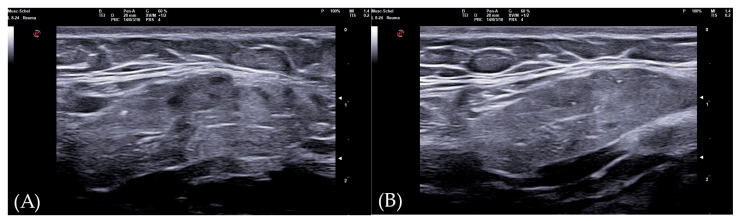
Submandibular glands in seronegative primary Sjogren syndrome. Female 57-year-old patient with peripheral symmetrical synovitis (some proximal inter-phalangeal and metacarpal-phalangeal of hands), sicca syndrome with positive Schirmer test, negative autoimmunity (ANA−, Ro-SSA−, La-SSB−, rheumatoid factor/ACPA−), positive labial biopsy for Sjogren syndrome, and type 1 diabetes mellitus. A coronal oblique scan over submandibular glands (8–24 MHz linear probe) shows a mild asymmetry on SGUS appearance: in the left side (**A**), moderate inhomogeneity with some hypo-anechoic areas occupying the superficial portion of gland (SGUS-OMERACT score = 2), whereas in the right side (**B**) SGUS shows mild inhomogeneity of the submandibular gland without hypo-anechoic areas (SGUS-OMERACT score = 1). The SGUS score of parotids was 2 on both sides. In this seronegative patient, only labial biopsy could define Sjogren syndrome diagnosis, but its SGUS global score of 7 had a 100% of specificity for the diagnosis.

The diagnostic accuracy of the global SGUS score (sum of SGUS scores of all four glands) to diagnose SS was comparable with those of the parotid and submandibular glands on one side. A mild SGUS asymmetry between the two sides can be observed (Figure 5). Moreover, as some patients could have unilateral complaints or show focal glandular enlargement with high risk of lymphoma, a global view of all four glands should be preferred. However, SGUS in only one side might be taken in consideration in the case of previous mono-lateral surgery or radiotherapy without reducing diagnostic accuracy. 

The correlation on SGUS scores between different types of salivary gland showed slightly worse values, as submandibular normal glands have a higher sono-structural inhomogeneity, and a score of one could be assigned also in non-pathologic conditions. This aspect has been underlined in previous works [14,15,18].

Our results show a reduced sensitivity (32.4%) (but higher specificity, 96.84%) in comparison with similar studies (sensitivity between 72% and 78%, specificity about 91%) [13,14]. This is likely related to the availability of labial biopsy in 100% of patients of our cohort, whereas in previous studies, a labial biopsy was performed in 34% [14] and 21% [13] of cases, leading to variations in the diagnostic gold standard. Moreover, the relatively short disease duration of our SS patients (59.5% with symptoms lasting for less of 3 years) could contribute to this lower sensitivity. 

In fact, the work of Zhang [14] suggests a lower sensitivity of SGUS in early phases of disease, while others demonstrate stability of gland abnormalities over time [19]. In other studies, disease duration correlates with the degree of gland atrophy [20]. Our data show more frequent diagnostic SGUS in longer disease duration. In any case, SS may remain asymptomatic for many years, and thus exert considerable difficulties on interpreting the real time of disease onset. Further studies should be addressed to clarify the accuracy of SGUS in the very early phases of disease.

Labial biopsy and autoantibodies have the highest weight in the ACR/EULAR classification criteria, whereas Schirmer test is a minor criterion. Labial biopsy is an invasive procedure, and it should be limited to as few patients as possible. Nevertheless, in the study of Fana et al. [15], 20% of their Ro-SSA-seronegative patients have a positive biopsy, and in 46% of patients the biopsy lead to a different disease classification. The Schirmer test lacks specificity for SS, as it constitutes a functional test that can be altered also in secondary forms of SS. 

A relevant aspect of our study is that labial biopsy was performed in all patients, and if the patient had a positive biopsy and at least one positive functional test, the diagnosis of SS was confirmed, independently from other criteria, in particular Ro-SSA status [5].

In our study, all Ro-SSA-seronegative sicca syndrome patients were correctly classified on SGUS as SS, without a false positive. On the other hand, eleven subject Ro-SSA+ had a labial biopsy negative for SS, and they constituted false positives that could be classified as SS using 2016 ACR/EULAR criteria. In our population, the proportion of Ro-SSA-seropositive SS patients was 46%, in line with recent studies [19]. The present study is limited by the retrospective nature of the design; in fact, we have no complete data on the positivity of La-SSB antibodies and the rheumatoid factor. Therefore, we cannot better clinically describe seronegative patients, as it was achieved in the study of Chatzis et al. [21].

Our large single-centre retrospective study confirms that SS is a pathology that mostly affects women (89%) between 40 and 60 years old, but it constitutes only less of a third (37/132, 28% in the present cohort) of patients referring with sicca syndrome. On the other hand, sicca syndrome is a condition that comprises about a quarter of subjects in which dryness constitutes only a referred symptom without glandular functional impairment or histological damages (34/132, 25.7% in our cohort) [22]. 

When our overall population is divided into three clinical phenotypes (Group 1 = subjective sicca syndrome, Group 2 = objective non-autoimmune sicca syndrome, Group 3 = objective autoimmune sicca syndrome), SGUS shows only one false positive in Group 1, and two false negatives in Group 3, confirming a high specificity of the examination (Table 2). In real clinical practice, SGUS seems to be useful in Group 1, where all the patients with positive biopsy are also correctly diagnosed by SGUS. Therefore, in the cases with subjective sicca syndrome and negative autoimmunity, a negative SGUS could conclude the diagnostic pathway, avoiding biopsy, as underlined in a previous work [23]. 

On the other hand, in Group 3, where autoimmunity is associated to objective sicca syndrome (satisfying ACR/EULAR criteria), SGUS can be used as a diagnostic confirmation. In these cases, if SGUS is positive, biopsy could be avoided or performed only to define immunological phenotypes [24].

In Group 2, our data suggest performing a complete diagnostic pathway comprising SGUS and biopsy.

As also reported in previous works, the OMERACT SGUS scoring system has demonstrated to correlate to functional status of salivary glands and serological biomarkers [25,26] 

In this scenario, it should be finally noted that SGUS is an inexpensive, non-invasive, and repeatable test. This could potentially obviate the need for biopsy in patients with “subjective” sicca syndrome, as suggested in recent works [27]. 

Furthermore, as it is not always possible to carry out a biopsy, our study highlights how with an SGUS score ≥ 7 it is possible to make a diagnosis of SS without a false positive, independently from the Ro-SSA status. 

This study has several limitations. Specifically, the study was conducted in a context of real clinical practice, so an assessment of intra- and inter-operator agreement is lacking. However, our diagnostic performance data are comparable with previous results [13,14,28]. Due to the same reasons, another limitation is lack of complete immunological data of SS patients. Moreover, no patient was classified as affected from secondary SS, preventing us from evaluating the diagnostic performances of SGUS in this subset. The only patient with peripheral synovitis (see Figure 5) could not be diagnosed with anything other than primary SS. In any case, previous studies demonstrated no significant difference in SGUS appearance between primary and secondary forms of SS [29].

Further studies should be carried out in order to correlate SGUS with autoimmunity status other than Ro-SSA and to clarify whether adipose gland involution could contribute to determining a hyposecretion, especially in older-aged patients with sicca syndrome.

## 5. Conclusions

This large mono-centre retrospective study confirms that SGUS, using the novel OMERACT scoring system, has moderate sensitivity and high specificity for the diagnosis of SS with good feasibility. The high specificity of this method could be useful for supporting the diagnosis of SS in cases of ANA seronegative or unavailability of biopsy. Moreover, SGUS should be considered as first-level method in the diagnostic pathway of patients with sicca syndrome, avoiding performing more invasive procedures in sicca syndrome patients seronegative for Ro-SSA with normal or unclear functional tests. 

## Figures and Tables

**Table 1:** Characteristics of study population. The initial cohort of patients with suspected sicca syndrome is indicated as “overall sicca syndrome”, whereas the subsequent columns describe patients with or without final diagnosis of primary Sjogren syndrome. SD = standard deviation, FS = focus score, SGUS = salivary glands ultrasonography.

	Overall Sicca Syndrome (132 Patients)	Primary Sjogren Syndrome (37 Patients)	Non Sjogren Syndrome (95 Patients)	Statistical Significance *p*-Value
Mean age, years, (±SD)	57.2 (SD 13.2)	57.4 (SD 14.7)	57.1 (SD 12.6)	n.s. 0.917
Female	93% (124/132)	89% (33/37)	96% (91/95)	n.s. 0.156
Schirmer ≤5 mm/5 min	70% (92/132)	76% (28/37)	67% (64/95)	n.s. 0.355
Anti-SSA positive	23% (31/132)	46% (17/37)	15% (14/95)	<0.001
FS ≥ 1 in labial gland biopsy	28% (37/132)	100% (37/37)	0% (0/95)	<0.001
Right side SGUS (±SD)	1.1 (1.35) range 0–6	1.97 (1.76) range 0–6	0.758 (0.975) range 0–6	<0.01
Left side SGUS (±SD)	0.902 (1.22) range 0–6	1.76 (1.64) range 0–6	0.568 (0.808) range 0–6	<0.01
Global SGUS (±SD)	2 (2.48) range 0–10	3.73 (3.29) range 0–10	1.33 (1.67) range 0–8	<0.01
Gland fibrosis	0.242 (0.732) range 0–4	0.514 (0.989) range 0–4	0.137 (0.576) range 0–4	0.035
Adipose gland involution	0.773 (1.03) range 0–4	0.784 (1.06) range 0–4	0.768 (1.03) range 0–4	n.s. 0.939
Lymph nodes	2.60 (1.74) range 0–7	2.87 (2.33) range 0–7	2.49 (1.44) range 0–7	n.s. 0.483

**Table 2:** Frequencies of positivity of biopsy and SGUS among the three clinical phenotype groups Group 1 = subjective sicca syndrome (positive subjective symptoms, negative functional testing and autoimmunity), Group 2 = objective non-autoimmune sicca syndrome (positive subjective symptoms and functional testing, negative autoimmunity), Group 3 = objective autoimmune sicca syndrome (positive subjective symptoms, functional testing, and autoimmunity).

	Overall Population	Final SS Diagnosis	Positive Biopsy	Positive SGUS
Group 1—subjective sicca syndrome	34	3	3	4
Group 2—objective non-autoimmune sicca syndrome	79	23	23	14
Group 3—objective autoimmune sicca syndrome	19	11	11	9

## Data Availability

Data are contained within the article.
